# *N*-(4-Chloro­phen­yl)-2-(hydroxy­imino)acetamide

**DOI:** 10.1107/S1600536809033315

**Published:** 2009-08-26

**Authors:** Jie Sun, Zaisheng Cai

**Affiliations:** a College of Chemistry, Chemical Engineering and Biotechnology, Donghua University, North Renmin Road No. 2999 Songjiang, Shanghai 201620, People’s Republic of China

## Abstract

The title compound, C_8_H_7_ClN_2_O_2_, is an inter­mediate in the synthesis of 5-chloro­isatin, which can be further transformed to 5-chloro-2-indolinone *via* a Wolff–Kishne reduction. The C_2_N acetamide plane forms a dihedral angle of 6.3 (3)° with the benzene ring. An intra­molecular C—H⋯O inter­action results in the formation of a six-membered ring. In the crystal, inter­molecular N—H⋯O, N—H⋯N and O—H⋯O hydrogen bonds link the mol­ecules into multimers, forming sheets.

## Related literature

For related structures, see: Miravitlles *et al.* (1974 ▶); Brianso *et al.* (1973 ▶); Liu *et al.* (2006 ▶). For the synthesis, see: Lai *et al.* (2003 ▶); Simon *et al.* (1997 ▶). 
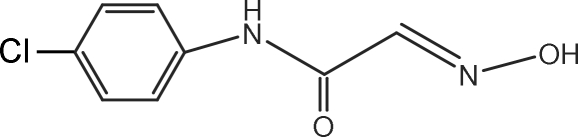


## Experimental

### 

#### Crystal data

C_8_H_7_ClN_2_O_2_
*M* *_r_* = 198.61
Orthorhombic, 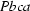

*a* = 10.101 (2) Å
*b* = 8.9150 (18) Å
*c* = 20.009 (4) Å
*V* = 1801.8 (6) Å^3^
*Z* = 8
Mo *K*α radiation
μ = 0.39 mm^−1^
*T* = 293 K
0.30 × 0.20 × 0.10 mm

#### Data collection

Enraf–Nonius CAD-4 diffractometer
Absorption correction: ψ scan (North *et al.*, 1968 ▶) *T*_min_ = 0.892, *T*_max_ = 0.962
3213 measured reflections
1639 independent reflections
1250 reflections with *I* > 2σ(*I*)
*R*_int_ = 0.031
3 standard reflections every 200 reflections intensity decay: 1%

#### Refinement

*R*[*F* ^2^ > 2σ(*F* ^2^)] = 0.047
*wR*(*F* ^2^) = 0.153
*S* = 1.00
1639 reflections
119 parameters
H-atom parameters constrained
Δρ_max_ = 0.40 e Å^−3^
Δρ_min_ = −0.36 e Å^−3^

### 

Data collection: *CAD-4 Software* (Enraf–Nonius, 1989 ▶); cell refinement: *CAD-4 Software*; data reduction: *XCAD4* (Harms & Wocadlo,1995 ▶); program(s) used to solve structure: *SHELXS97* (Sheldrick, 2008 ▶); program(s) used to refine structure: *SHELXL97* (Sheldrick, 2008 ▶); molecular graphics: *PLATON* (Spek, 2009 ▶); software used to prepare material for publication: *SHELXL97* and *PLATON*.

## Supplementary Material

Crystal structure: contains datablocks global, I. DOI: 10.1107/S1600536809033315/fl2255sup1.cif

Structure factors: contains datablocks I. DOI: 10.1107/S1600536809033315/fl2255Isup2.hkl

Additional supplementary materials:  crystallographic information; 3D view; checkCIF report

## Figures and Tables

**Table 1:** Hydrogen-bond geometry (Å, °)

*D*—H⋯*A*	*D*—H	H⋯*A*	*D*⋯*A*	*D*—H⋯*A*
N1—H1*A*⋯O1^i^	0.86	2.52	3.115 (3)	127
N1—H1*A*⋯N2^i^	0.86	2.31	3.140 (3)	163
O2—H2*A*⋯O1^ii^	0.82	1.98	2.785 (3)	167
C5—H5*A*⋯O1	0.93	2.32	2.918 (3)	122

Symmetry codes: (i) 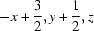; (ii) 
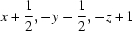
.

**Fig. 1.**
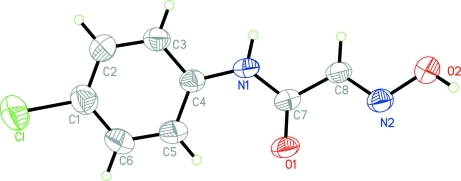
The molecular structure of the title molecule, with the atom-numbering scheme. Displacement ellipsoids are drawn at the 50% probability level. Hydrogen bond is shown as dashed line.

**Fig. 2.**
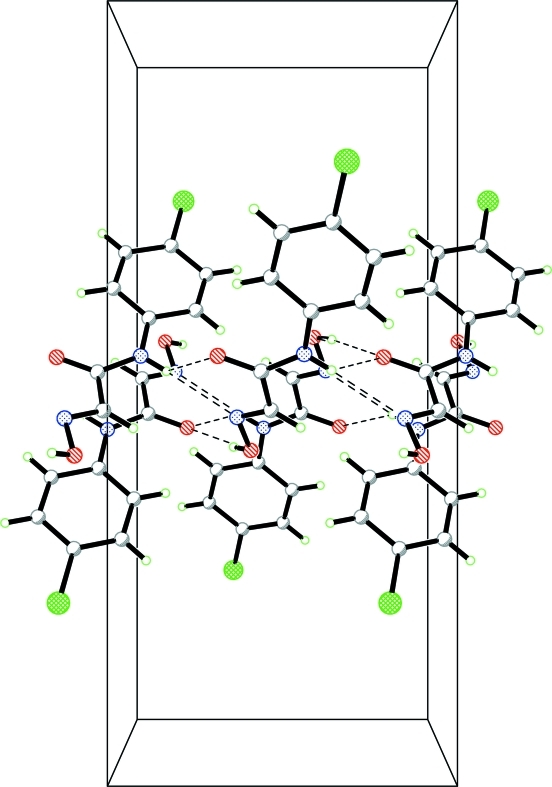
A partial packing diagram of the title compound. Hydrogen bonds are shown as dashed lines.

## supplementary crystallographic information

### Comment

The title compound is an important intermediate in the synthesis of 5-chloro-isatin,which can be further transformed to 5-chloro-2-indolinone *via* a Wolff-Kishne reduction.

As part of our ongoing studies on phenyl-substituted-2-indolinone(Lai *et al.*, 2003; Simon *et al.*,1997), the crystal structure of (*E*)—*N*-(2-chlorophenyl)-2-(hydroxyimino)acetamide and (*E*)-2-(hydroxyimino)-*N*-phenylacetamide have been reported(Miravitlles *et al.*,1974; Brianso *et al.*,1973; Liu *et al.*,2006), we report herein the crystal structure of the title compound.

In the title compound (Fig 1), the bond lengths and angles are within normal ranges. The central acetamide plane N1/C7/O1/C8 forms a dihedral angle of 6.3 (3)° with the phenyl ring. An intramolecular C—H···O interaction results in the formation of a six-membered ring. In the crystal packiing, intermolecular N—H···O and N—H···N hydrogen bonds (Table 1) link the molecules into multimers (Fig. 2), ithat may be effective in the stabilization of the structure.

### Experimental

85 g (0.06 mol) sodium sulfate and 300 ml water were added to a 1000 ml 3 mouthed flask, mixed until the sodium sulfate dissolved following which a saturated solution of 18 g (0.11 mol) chloral hydrate was added. While stirring, a mixture of 12.7 g(0.1 mol) *p*-chloroaniline, 12 ml hydrochloric acid and 100 ml water was added dropwise causing a white precipitate. Then 22 g(0.32 mol) hydroxylamine hydrochloride was added and the mixture was heated to 348k. After 5 h, a light yellow precipitate appeared which was filtered and washed with water, dried and recrystallized from ethanol (yield 90.2%). Crystals suitable for X-ray analysis were obtained by slow evaporation of an acetone solution (yield; 90%, m.p. 443 K).

### Refinement

H atoms were positioned geometrically, with O—H = 0.82 Å (for OH), N—H=0.86Å (for NH) and C—H =0.93Å for aromatic and methylene H, respectively, and constrained to ride on their parent atoms, with *U*_iso_(H) = *xU*_eq_(C,*O*,*N*), where *x* = 1.5 for OH H and *x* = 1.2 for all other H atoms.

### Crystal data

**Table:**

C_8_H_7_ClN_2_O_2_	*D*_x_ = 1.464 Mg m^−^^3^
*M**_r_* = 198.61	Melting point: 443 K
Orthorhombic, *P**b**c**a*	Mo *K*α radiation, λ = 0.71073 Å
Hall symbol: -P 2ac 2ab	Cell parameters from 25 reflections
*a* = 10.101 (2) Å	θ = 10–14°
*b* = 8.9150 (18) Å	µ = 0.39 mm^−^^1^
*c* = 20.009 (4) Å	*T* = 293 K
*V* = 1801.8 (6) Å^3^	Block, yellow
*Z* = 8	0.30 × 0.20 × 0.10 mm
*F*(000) = 816	

### Data collection

**Table:**

Enraf–Nonius CAD-4 diffractometer	1250 reflections with *I* > 2σ(*I*)
Radiation source: fine-focus sealed tube	*R*_int_ = 0.031
graphite	θ_max_ = 25.3°, θ_min_ = 2.0°
ω/2θ scans	*h* = 0→12
Absorption correction: ψ scan (North *et al.*, 1968)	*k* = 0→10
*T*_min_ = 0.892, *T*_max_ = 0.962	*l* = −24→24
3213 measured reflections	3 standard reflections every 200 reflections
1639 independent reflections	intensity decay: 1%

### Refinement

**Table:**

Refinement on *F*^2^	Secondary atom site location: difference Fourier map
Least-squares matrix: full	Hydrogen site location: inferred from neighbouring sites
*R*[*F*^2^ > 2σ(*F*^2^)] = 0.047	H-atom parameters constrained
*wR*(*F*^2^) = 0.153	*w* = 1/[σ^2^(*F*_o_^2^) + (0.1*P*)^2^ + 0.25*P*] where *P* = (*F*_o_^2^ + 2*F*_c_^2^)/3
*S* = 1.00	(Δ/σ)_max_ < 0.001
1639 reflections	Δρ_max_ = 0.40 e Å^−^^3^
119 parameters	Δρ_min_ = −0.36 e Å^−^^3^
0 restraints	Extinction correction: *SHELXL97* (Sheldrick, 2008), Fc^*^=kFc[1+0.001xFc^2^λ^3^/sin(2θ)]^-1/4^
Primary atom site location: structure-invariant direct methods	Extinction coefficient: 0.013 (3)

### Special details

**Table:**

Geometry. All e.s.d.'s (except the e.s.d. in the dihedral angle between two l.s. planes) are estimated using the full covariance matrix. The cell e.s.d.'s are taken into account individually in the estimation of e.s.d.'s in distances, angles and torsion angles; correlations between e.s.d.'s in cell parameters are only used when they are defined by crystal symmetry. An approximate (isotropic) treatment of cell e.s.d.'s is used for estimating e.s.d.'s involving l.s. planes.
Refinement. Refinement of *F*^2^ against ALL reflections. The weighted *R*-factor *wR* and goodness of fit *S* are based on *F*^2^, conventional *R*-factors *R* are based on *F*, with *F* set to zero for negative *F*^2^. The threshold expression of *F*^2^ > σ(*F*^2^) is used only for calculating *R*-factors(gt) *etc*. and is not relevant to the choice of reflections for refinement. *R*-factors based on *F*^2^ are statistically about twice as large as those based on *F*, and *R*- factors based on ALL data will be even larger.

### Fractional atomic coordinates and isotropic or equivalent isotropic displacement parameters (Å^2^)

**Table:**

	*x*	*y*	*z*	*U*_iso_*/*U*_eq_	
Cl	0.26935 (10)	0.17533 (11)	0.78148 (5)	0.0896 (4)	
O1	0.65722 (17)	−0.19066 (16)	0.54941 (10)	0.0497 (5)	
N1	0.62352 (19)	0.0637 (2)	0.55162 (10)	0.0425 (5)	
H1A	0.6442	0.1442	0.5304	0.051*	
C1	0.3696 (3)	0.1410 (3)	0.71252 (15)	0.0565 (7)	
O2	0.96195 (19)	−0.0993 (2)	0.42075 (9)	0.0572 (6)	
H2A	1.0264	−0.1546	0.4246	0.086*	
N2	0.86646 (19)	−0.1394 (2)	0.46737 (10)	0.0435 (5)	
C2	0.4060 (3)	0.2563 (3)	0.67015 (13)	0.0571 (7)	
H2C	0.3749	0.3531	0.6775	0.068*	
C3	0.4883 (2)	0.2267 (3)	0.61720 (13)	0.0473 (6)	
H3A	0.5137	0.3044	0.5889	0.057*	
C4	0.5342 (2)	0.0825 (2)	0.60529 (12)	0.0392 (6)	
C5	0.4961 (3)	−0.0326 (3)	0.64785 (13)	0.0572 (8)	
H5A	0.5260	−0.1298	0.6405	0.069*	
C6	0.4138 (3)	−0.0022 (3)	0.70111 (15)	0.0610 (8)	
H6A	0.3881	−0.0794	0.7295	0.073*	
C7	0.6807 (2)	−0.0630 (2)	0.52908 (12)	0.0393 (6)	
C8	0.7835 (2)	−0.0349 (3)	0.47759 (12)	0.0415 (6)	
H8A	0.7870	0.0549	0.4540	0.050*	

### Atomic displacement parameters (Å^2^)

**Table:**

	*U*^11^	*U*^22^	*U*^33^	*U*^12^	*U*^13^	*U*^23^
Cl	0.0956 (7)	0.0920 (7)	0.0814 (6)	0.0178 (5)	0.0434 (5)	0.0147 (5)
O1	0.0536 (11)	0.0290 (9)	0.0667 (11)	−0.0014 (7)	0.0034 (9)	0.0062 (8)
N1	0.0494 (11)	0.0265 (9)	0.0518 (12)	−0.0012 (8)	0.0038 (10)	0.0060 (8)
C1	0.0499 (15)	0.0637 (17)	0.0559 (16)	0.0043 (13)	0.0082 (12)	0.0062 (13)
O2	0.0517 (11)	0.0475 (10)	0.0725 (13)	0.0092 (9)	0.0147 (10)	0.0054 (9)
N2	0.0431 (11)	0.0347 (10)	0.0526 (12)	−0.0002 (9)	0.0000 (9)	0.0023 (9)
C2	0.0609 (17)	0.0482 (14)	0.0621 (17)	0.0131 (13)	0.0106 (14)	0.0045 (13)
C3	0.0477 (14)	0.0404 (13)	0.0538 (14)	0.0021 (11)	0.0040 (12)	0.0088 (11)
C4	0.0387 (12)	0.0340 (12)	0.0449 (12)	−0.0032 (10)	−0.0013 (10)	0.0016 (10)
C5	0.0754 (19)	0.0340 (13)	0.0621 (16)	−0.0052 (13)	0.0115 (15)	0.0025 (12)
C6	0.0713 (19)	0.0507 (15)	0.0611 (16)	−0.0100 (14)	0.0161 (14)	0.0107 (14)
C7	0.0394 (12)	0.0311 (11)	0.0472 (13)	0.0000 (10)	−0.0072 (11)	0.0024 (9)
C8	0.0453 (13)	0.0303 (11)	0.0491 (13)	0.0018 (10)	−0.0003 (10)	0.0048 (10)

### Geometric parameters (Å, °)

**Table:**

Cl—C1	1.739 (3)	C2—C3	1.373 (3)
O1—C7	1.232 (3)	C2—H2C	0.9300
N1—C7	1.346 (3)	C3—C4	1.387 (3)
N1—C4	1.412 (3)	C3—H3A	0.9300
N1—H1A	0.8600	C4—C5	1.388 (3)
C1—C6	1.371 (4)	C5—C6	1.378 (4)
C1—C2	1.382 (4)	C5—H5A	0.9300
O2—N2	1.389 (3)	C6—H6A	0.9300
O2—H2A	0.8200	C7—C8	1.484 (3)
N2—C8	1.270 (3)	C8—H8A	0.9300
			
C7—N1—C4	128.95 (19)	C3—C4—N1	117.01 (19)
C7—N1—H1A	115.5	C5—C4—N1	123.8 (2)
C4—N1—H1A	115.5	C6—C5—C4	119.8 (2)
C6—C1—C2	120.2 (3)	C6—C5—H5A	120.1
C6—C1—Cl	119.1 (2)	C4—C5—H5A	120.1
C2—C1—Cl	120.7 (2)	C1—C6—C5	120.5 (2)
N2—O2—H2A	109.5	C1—C6—H6A	119.7
C8—N2—O2	112.20 (19)	C5—C6—H6A	119.7
C3—C2—C1	119.5 (3)	O1—C7—N1	125.6 (2)
C3—C2—H2C	120.3	O1—C7—C8	121.3 (2)
C1—C2—H2C	120.3	N1—C7—C8	113.04 (19)
C2—C3—C4	120.9 (2)	N2—C8—C7	116.7 (2)
C2—C3—H3A	119.6	N2—C8—H8A	121.6
C4—C3—H3A	119.6	C7—C8—H8A	121.6
C3—C4—C5	119.1 (2)		
			
C6—C1—C2—C3	−1.0 (4)	C2—C1—C6—C5	0.8 (5)
Cl—C1—C2—C3	178.1 (2)	Cl—C1—C6—C5	−178.4 (2)
C1—C2—C3—C4	0.7 (4)	C4—C5—C6—C1	−0.2 (4)
C2—C3—C4—C5	−0.2 (4)	C4—N1—C7—O1	5.3 (4)
C2—C3—C4—N1	−177.1 (2)	C4—N1—C7—C8	−171.9 (2)
C7—N1—C4—C3	−179.0 (2)	O2—N2—C8—C7	−177.10 (19)
C7—N1—C4—C5	4.3 (4)	O1—C7—C8—N2	−16.5 (3)
C3—C4—C5—C6	−0.1 (4)	N1—C7—C8—N2	160.9 (2)
N1—C4—C5—C6	176.6 (2)		

### Hydrogen-bond geometry (Å, °)

**Table:**

*D*—H···*A*	*D*—H	H···*A*	*D*···*A*	*D*—H···*A*
N1—H1A···O1^i^	0.86	2.52	3.115 (3)	127
N1—H1A···N2^i^	0.86	2.31	3.140 (3)	163
O2—H2A···O1^ii^	0.82	1.98	2.785 (3)	167
C5—H5A···O1	0.93	2.32	2.918 (3)	122

Symmetry codes: (i) −*x*+3/2, *y*+1/2, *z*; (ii) *x*+1/2, −*y*−1/2, −*z*+1.

## References

[bb1] Brianso, J. L., Miravitlles, C., Font-Altaba, M., Declercq, J. P. & Germain, G. (1973). *Cryst. Struct. Commun.* **2**, 319–321.

[bb2] Enraf–Nonius (1989). *CAD-4 Software* Enraf–Nonius, Delft. The Netherlands.

[bb3] Harms, K. & Wocadlo, S. (1995). *XCAD4* University of Marburg, Germany.

[bb4] Lai, Y., Zhang, Y. & Li, Y. (2003). *Zhongguo Yaowu Huaxue Zazhi*, **13**, 99–101.

[bb5] Liu, S., Ma, M., Zhou, H., Li, Y. & Han, L. (2006). *Acta Cryst.* E**62**, o316–o317.

[bb6] Miravitlles, C., Plana, F., Brianso, J. L. & Font-Altaba, M. (1974). *Cryst. Struct. Commun.* **3**, 439–442.

[bb7] North, A. C. T., Phillips, D. C. & Mathews, F. S. (1968). *Acta Cryst.* A**24**, 351–359.

[bb8] Sheldrick, G. M. (2008). *Acta Cryst.* A**64**, 112–122. 10.1107/S0108767307043930 18156677

[bb9] Simon, J. G., Jose, C. T., Alexandre, A. F., Rosangela, B. S. & Angelo, C. P. (1997). *Tetrahedron Lett.* **38**, 1501–1504.

[bb10] Spek, A. L. (2009). *Acta Cryst.* D**65**, 148–155. 10.1107/S090744490804362X PMC2631630 19171970

